# Ethical Considerations for Movement Mapping to Identify Disease Transmission Hotspots

**DOI:** 10.3201/eid2507.181421

**Published:** 2019-07

**Authors:** Bouke C. de Jong, Badou M. Gaye, Jeroen Luyten, Bart van Buitenen, Emmanuel André, Conor J. Meehan, Cian O’Siochain, Kristyna Tomsu, Jérôme Urbain, Koen Peeters Grietens, Maureen Njue, Wim Pinxten, Florian Gehre, Ousman Nyan, Anne Buvé, Anna Roca, Raffaella Ravinetto, Martin Antonio

**Affiliations:** Institute of Tropical Medicine, Antwerp, Belgium (B.C. de Jong, C.J. Meehan, K.P. Grietens, M. Njue, F. Gehre, A. Buvé, R. Ravinetto);; Medical Research Council Unit, The Gambia at London School of Hygiene & Tropical Medicine, Fajara, The Gambia (B.M. Gaye, C. O’Siochain, F. Gehre, A. Roca, M. Antonio);; Katholieke Universiteit Leuven, Leuven, Belgium (J. Luyten, E. André);; White Wire Data Protection, Kontich, Belgium (B. van Buitenen);; Dalberg Data Insights, Brussels, Belgium (K. Tomsu, J. Urbain);; School of Tropical Medicine and Global Health, Nagasaki University, Nagasaki, Japan, and Amsterdam Institute for Social Science Research, Amsterdam, the Netherlands (K.P. Grietens);; Hasselt University, Hasselt, Belgium (W. Pinxten);; Bernhard Nocht Institute for Tropical Medicine, Hamburg, Germany (F. Gehre);; University of The Gambia, Serrekunda, The Gambia (O. Nyan)

**Keywords:** ethics, transmission hotspots, movement mapping, infectious diseases, GPS, smartphones, mobile phones

## Abstract

Traditional public health methods for detecting infectious disease transmission, such as contact tracing and molecular epidemiology, are time-consuming and costly. Information and communication technologies, such as global positioning systems, smartphones, and mobile phones, offer opportunities for novel approaches to identifying transmission hotspots. However, mapping the movements of potentially infected persons comes with ethical challenges. During an interdisciplinary meeting of researchers, ethicists, data security specialists, information and communication technology experts, epidemiologists, microbiologists, and others, we arrived at suggestions to mitigate the ethical concerns of movement mapping. These suggestions include a template Data Protection Impact Assessment that follows European Union General Data Protection Regulations.

Human and pathogen co-evolution has led to a vast array of transmission routes, transmission dynamics, and risks for infection. Human behavior, particularly movement between locations, plays a primary role in the transmission dynamics of infectious diseases (*1*). Geospatial areas with high prevalence or efficient transmission of disease are known as hotspots (*2*). Transmission hotspots can be thought of as nodes in space and time where the density of contact between infected and uninfected persons is higher than average, increasing the risk for disease transmission. 

Interrupting transmission is key to preventing and controlling infectious diseases (*1*). Timely identification of transmission routes and hotspots is necessary for tailoring public health interventions. However, many interventions that effectively break transmission chains also invade the private sphere of affected persons or communities and often are at odds with personal liberties (*3*). Some methods of identifying transmission hotspots could reveal sensitive information on behavior and the inner functioning of a community, making these methods problematic from an ethics point of view.

Traditional public health approaches to mapping human-to-human transmission routes include contact tracing and molecular epidemiology. Both approaches are laborious, costly, and limited. Contact tracing, in which investigators follow up with named contacts to identify those at risk for exposure (*4*), lacks sensitivity for population-based interruption of transmission chains. Molecular epidemiology uses genetic typing of pathogens isolated from patients to trace transmission by highlighting genetic similarity (*5*) but is prone to undersampling, potentially missing key events in complex transmission chains. Because of laboratory delays, molecular epidemiology frequently does not lead to actionable findings. In addition, this method is not feasible outside of research institutions in many low- and middle-income countries, where higher rates of infectious diseases occur.

A promising alternative to contact tracing is to identify transmission hotspots where public health workers can tailor preventive strategies. During an infectious disease outbreak, as control increases in the general population, the disease typically spreads heterogeneously. Transmission events then concentrate in areas and communities not reached by conventional approaches. 

When the goal is elimination and ultimate eradication of a specific disease, tackling transmission hotspots is key. Information and communication technology (ICT), such as global positioning systems (GPS), smartphones, and mobile phones, could provide a novel approach to identifying transmission hotspots (Appendix 1). One promising approach is to map movements of persons by using ICT data to identify behavior patterns and transmission hotspots where cost-effective prevention strategies could be implemented. This type of mapping could reduce transmission of diseases that are difficult to eliminate, such as tuberculosis (TB), leprosy, schistosomiasis, malaria (*6*), and sleeping sickness, and assist in controlling outbreaks of acute disease, such as Ebola virus (*7*,*8*), cholera, or Shiga toxin–producing *Escherichia coli* O157:H7 (*9*). However, mapping the movements of potentially infected persons comes with many ethical challenges.

We proposed a project in which researchers request informed consent from TB patients to map their aggregate movements through their cellular phone call detail records (CDRs). In the context of this project, we convened a 1-day meeting on the ethical aspects around the use of mobility data for mapping infectious disease transmission. The meeting, held October 24, 2017, at the Institute of Tropical Medicine (Antwerp, Belgium), included researchers, ethicists, data security specialists, ICT experts, epidemiologists, microbiologists, and a representative from a national TB program (Appendix 2). The objective for the 20 participants was to consider risks and benefits of using ICT data to map movements of infected persons and identify transmission hotspots of TB and other infectious diseases. From this meeting, we developed a model Data Protection Impact Assessment (DPIA) template that others can use to conduct a similar assessment (Appendix 3). We focused mainly on ethical aspects for research but also addressed specific concerns that could arise if the approach is scaled up for programmatic use.

## Data Sources for Detecting Transmission Hotspots

Information on case mobility, typically collected by public health officials or researchers through patient interviews, is crucial for tracking pathogen transmissions. However, the limited population sample interviewed introduces selection bias. In addition, questionnaire data on mobility might lack sufficient detail and are prone to both recall and information bias. Because of the complexity and cost, interviews and questionnaires are difficult to implement in programmatic conditions and do not allow for real-time interpretation using autonomous self-learning algorithms. 

The global penetration of mobile phones could provide a viable means to track infectious diseases by electronically mapping case mobility data. Worldwide, 66% of the population used mobile phones in 2017, counting unique mobile subscribers and corrected for use of multiple subscriber identification module (SIM) cards. Of global SIM cards, 57% are used in smartphones, without correcting for multiple SIM card users (*10*). We discussed 3 options for collecting ICT data: a dedicated smartphone application, a separate GPS tracking device, and mobile phone call records (Table; Appendix 1).

**Table:** Characteristics of different approaches to collecting mobility data for mapping infectious disease outbreaks*

Characteristics	Source of mobility data		
	Dedicated smartphone application	GPS tracker	Call detail records
Scalability to large populations	Medium	Low	High
Retrospective analysis possible	Likely, depending on stored location data on phone at time of installation	No	Likely, depending on duration of data storage at telecom operators
Spatial resolution	High, depending on mobile data use and WiFi density	High	Variable, depending on cell tower and mast density
Participant control	Medium	High	Low
Third party access to private information	Possibly	Unlikely	Likely
Need for uninfected controls	Possibly	Unlikely	Likely, to avoid identification of health information by telecom operators

*GPS, global positioning system.

## Ethical Issues of Mobility Mapping

Collecting mobility data linked to health information poses specific challenges for upholding ethics principles described in basic guidelines for human subjects research. We see 2 highly relevant ethical obstacles: protecting the participants’ privacy in relation to principles of autonomy and nonmaleficence and finding a balance between costs, risks, and benefits for participants and communities in relation to principles of beneficence and justice (*11*).

## Protecting Participants’ Privacy

The European Union General Data Protection Regulation (EU GDPR, EU Regulation 2016/679) (*12*) and Guideline 22 of the Council for International Organizations of Medical Sciences (*13*), among other regulations, explicitly stress the need for protecting the privacy and confidentiality of persons and their information. In all situations, investigators must put protective measures in place to avoid breaches in confidential mobility data that might cause unintended inferences about a user’s life (*14*).

Outbreaks of highly infectious diseases, such as Ebola and cholera, along with endemic diseases, such as HIV and TB, are more prevalent in low-income countries. Many mobility tracking approaches have been implemented to identify infectious disease hotspots in these areas (*6*,*15*–*19*). In international collaborations, researchers from abroad should apply the same ethics and regulatory standards they would apply in their own countries. EU-funded researchers working outside the EU must ensure their research adheres to EU GDPR standards. Mapping across national boundaries might require further ethics and data security safeguards and the perceived balance between benefit and risk of mapping mobility might be different in diverse social and cultural contexts.

In some circumstances, investigators might have difficulty meeting ethics requirements for informed consent. Waivers for informed consent can be granted by the concerned ethics committees, but only under exceptional circumstances; for instance, during outbreaks, if the research is low-risk and has potential for high societal value; if obtaining written consent is unpractical or unfeasible; and if data anonymization and other adequate measures are in place to mitigate confidentiality and privacy risks. 

Linking health conditions or diagnoses with patient movements constitutes a high risk to infected persons. Consequently, investigators should not have access to the movements of individual patients, and telecommunications staff should not be able to link movements of person, with their health-related information. Participants should be aware of these risks when asked for their telephone data. They also should be informed of their right to refuse or withdraw their consent for use of their data.

The challenges are even greater for mapping movements of children. Most children do not routinely keep mobile phones; great cultural and socioeconomic variations exist in the age of first phone use and in adult supervision. Children under a certain age presumably would be accompanied by adults. Just as in other kinds of research, minors should be asked for assent when their parent or guardian is asked for informed consent, according to their capacity and maturity, while adhering to local regulatory requirements. Additional ethics challenges might arise if parents or guardians request access to the mobility data of their children.

## Costs, Risks, and Benefits for Participants and Communities

In public health ethics, the dilemma of individual risk versus population benefit is well-recognized (*20*,*21*). When weighing risks to participants’ privacy against potential population benefits, researchers must consider the participants’ status. Patients, contacts of patients, or healthy persons sampled from the general population will have different risks to their privacy or benefits for their communities. 

Study participants will not directly benefit from knowing where they might have acquired or spread an infectious disease. Therefore, the realistic potential of a study to contribute to improved public health must be considerable to outweigh the risk to the participant. Spatiotemporal analysis of data can show where infected persons crossed paths when they were infectious but is not proof of actual transmission events. These data can indicate locations where the density of infected persons was higher and might point to previously unsuspected transmission hotspots.

Communities or neighborhoods with confirmed or suspected infectious disease transmission hotspots might be stigmatized. Such stigma could have further negative consequences, such as discrimination against groups or neighborhoods, reduced tourism (*22*), or decreased property values. Although anonymized data can focus on neighborhoods rather than specific buildings, stigma could occur at businesses, schools, social venues, or healthcare facilities in an area identified as a transmission hotspot. Researchers should consider such risks and plan adequate mitigating measures during protocol development and should include representatives from involved communities during protocol development. Community representatives can provide a firsthand understanding of local challenges, such as the community’s perception of the disease; whether specific persons are typically stigmatized in the community; whether persons could be legally prosecuted for behaviors associated with disease transmission, such as drug use or same sex intercourse in some areas; and how to provide information back to the community. Researchers should not presume that they can identify representative community leaders. In some contexts, leaders are obvious, such as patients with HIV or TB who are active in local associations, but identifying these leaders might be more difficult in urban settings or in disrupted communities under the stress of an outbreak.

In contrast to the ethical risks, researchers also should consider whether withholding the vast amount of mobility data would be unethical and whether an ethical imperative exists to use the available data for maximal benefit (*23*). Ultimately, if residual risk is acceptable, analysis of mobility data can be justified if it can yield actionable insights that benefit public health.

Researchers also should consider whether, and how, to communicate information on hotspots to the general population. In doing so, they must question whether public health interventions in the hotspots are sufficient to reduce the risk for infection for persons moving into and out of those areas. If so, avoiding communication about specific hotspots could reduce the chance for stigma in that community. On the other hand, if persons moving into or out of hotspots need to take protective measures, such as wearing a mask or avoiding the area altogether, then researchers should put the utmost care into communicating appropriate messages.

### Mitigating Ethical Challenges

During the protocol design phase, researchers should assess the risk-benefit balance of their intended movement mapping strategy, address risks for privacy breaches, and plan for mitigating such breaches. The Global System for Mobile Communication Association and others have developed guidelines and outlined ethical challenges for using telecommunication data (*24**–**27*). However, no single framework will fit the myriad of movement mapping approaches or all applications for the identification of transmission hotspots of infectious diseases.

According to the EU GDPR, personal data can only be processed under specific circumstances for a well-defined and communicated purpose, and only when participants consent and data processing is proportionate to the purpose (*12*). Personal data cannot be processed beyond what is known or expected by the research participant and cannot be kept longer than needed.

A DPIA provides a framework to mitigate risks by taking privacy principles into account in the earliest conception and engineering phases of a project or data processing application (Appendix 3). Along with an analysis of residual risks, researchers should conduct a DPIA before beginning data collection.

## Involving Communities as Research Stakeholders 

Including the community and its members as stakeholders during the design phase will help investigators convey the public health benefits of the project and minimize misunderstanding, mistrust, and panic. To maintain accountability to communities, researchers should involve them in the preparation, implementation, and evaluation of aggregate movement mapping data. By working with community members, investigators can address concerns about risks and benefits to the community and its members. 

Potential approaches to enhancing community input include involving community advisory boards or similar community structures in the study protocol design (*28*,*29*); carrying out a preliminary qualitative study to investigate the perception of local policy makers and members of the community about the mobility data collection; or carrying out a rapid ethics assessment, a brief qualitative intervention used to examine the ethics terrain of a research setting before recruiting participants (*30*). Such participatory research approaches engage communities in the research process and take local perception into account during research planning (*29*). 

To further reduce the chance of stigmatization of the neighborhoods or groups, researchers should install measures to prevent identification of transmission hotspot locations by outside parties. Such measures could include confidentiality agreements with local public health authorities and communication plans to reduce the consequences of misuse of information by the media or other actors.

## Aggregating Data

Researchers should use aggregate analyses of mobility data to reduce the risk of breaching participants’ privacy and confidentiality (*24*,*25*). This process could involve anonymizing phone numbers associated with CDRs so that patient information can be viewed without revealing identities or phone numbers of participants. In addition, researchers can use algorithms that permit aggregate analysis of a minimum number of participants’ CDRs to reduce the chance of pinpointing a participant’s movements. Aggregate CDRs provide background mobility controls, while still calculating relative risks of transmission hotspots. In the absence of aggregate CDRs, investigators can still identify hotspots with few relative risks to participants’ privacy.

## Requesting Informed Consent

Informed consent and patient information sheets should be transparent, specific, and unambiguous. The aim is to guide participants’ understanding of the project’s purpose, as well as how investigators will handle their personal data, protect their confidentiality, and address any residual risks to personal information after the study. During the consent interview or in the consent documentation, researchers should reduce jargon and present information in plain language appropriate for the audience. Investigators also might use teach-back methods, back translation, and pilot testing with the target group to assist in developing accurate informed consent and patient information sheets. 

Participants should be informed how long their data will be stored before destruction and the deadline by which they can request rectification or destruction of their data. A rapid ethics assessment also can help improve the design of the informed consent tools for a given population. Study personnel should be properly trained on the informed consent and data collection procedures. 

## Considerations for Telecommunications Regulatory Authorities and Providers

In addition to ethics approvals, the telecommunication regulatory authorities must approve use of CDRs and mobile phone companies will require confidentiality and data transfer agreements. Researchers will need to ensure that mobile network operators and their employees comply with the same confidentiality rules applied in the health sector, including securely handling, storing, and limiting access to data. Agreements with mobile phone companies should specify that they not share data with other parties or retain it longer than necessary.

One way to minimize the chance for mobile network employees or others to link CDRs to a particular disease profile is through recruiting uninfected participants as control cases. Researchers can include uninfected participants’ CDRs, with their consent, from the same catchment area as the patients. Control participants benefit public health without gaining a personal benefit, but they face the same risk for confidentiality breaches. In addition, such breaches could cause them to be mistaken as infected with the disease. No clear precedent exists to help set a dilution rate (i.e., the ratio of uninfected controls to patients) to sufficiently mitigate the chances of linking a person with an infectious disease diagnosis. However, more controls might favorably shift the risk-benefit balance.

For data analysis, researchers should use algorithms that prevent disaggregation of data and reduce opportunities to link health information and mobility patterns with any participant’s identity. After aggregating the analysis, investigators can filter out controls to arrive at hotspot information.

## Communicating Information on Transmission Hotspots

When researchers publish study findings, they should clearly demonstrate that they followed all ethics rules and discussed a response plan with local communities and public health authorities, especially when they discuss a previously unknown public health problem in a specific community. To avoid revealing exact locations, researchers can include graphical representations rather than recognizable maps. Researchers also can request to override any requirements for sharing the full or aggregated dataset and associated metadata at the time of publication due to privacy concerns. 

Regardless of safeguards, the media, authorities, or politicians could misuse published results or take the findings out of context. Although a data transfer agreement with a mobile network will not prevent misuse of information, researchers can reduce negative consequences of such misuse by developing a comprehensive communication plan with community stakeholders and public health authorities before starting the project.

Researchers should have an adequate understanding of the mobile technologies and related ethics requirements for each specific research project. Ethics committees might lack the knowledge and expertise to assess these protocols correctly and researchers should be prepared to explain the technologies and how they can benefit public health. In addition, ethics committees and institutional review boards should consider involving data security specialists as more research begins to incorporate mobile technologies.

## Implementing Mobile Mapping

After a research project demonstrates the feasibility of ethically using CDRs for identifying transmission hotspots, a national control program might choose to implement it. For a TB program, patients with confirmed TB could provide informed consent to release their CDRs to map their movements. Staff from the national TB program could periodically perform an aggregate analysis of recent CDRs to identify locations where transmission might have occurred. 

In addition to ethics issues tackled during research, public health programs could encounter more ethics complexities. For instance, patients might experience a therapeutic misconception and feel obliged to consent to releasing their CDRs if the request comes from the same physician who is providing healthcare because they fear care will be withheld if they do not consent. Also, programs should be aware that routine surveillance data could be used for retrospective research, blurring the boundary between surveillance and research. Even if such surveillance is not intended as research, a public health program should involve the appropriate ethics committee. The ethics committee can assess the protocol for collecting mobility data and the informed consent tool, as well as the proposed project’s public engagement and transparency plans to determine its benefit to the public (*31*).

## Conclusions

With their unprecedented global penetration, mobile phones can yield vast amounts of information that offer opportunities for mapping infectious disease hotspots but also pose ethical challenges. We offer suggestions on safeguards to ensure data can be used to benefit public health while protecting the users’ privacy and confidentiality. The EU GDPR protects EU residents and participants in EU-funded research in countries outside of the EU from abuse of personal information. The higher standards for the EU GDPR also apply to the ethics framework for research on mobility patterns conducted for public health benefits. By upholding these ethics standards, public health investigators could use mobility mapping to identify infectious disease transmission hotspots without compromising the privacy of patients or creating mistrust in communities affected by infectious diseases.

Appendix 1 Overview of approaches to collecting mobility data for mapping infectious disease outbreaks, including information on smartphones, global positioning system trackers, and call detail records.

Appendix 2 Background, objective, participants, and agenda for the 1-day workshop on the ethical aspects on the use of individuals’ mobility data for mapping infectious diseases held October 24, 2017, at the Institute of Tropical Medicine, Antwerp, Belgium.

Appendix 3 Data protection impact assessment template developed by White Wire Data Protection, Kontich, Belgium.
