# Supplementary material for: Ethical Considerations for Movement Mapping to Identify Disease Transmission Hotspots
Source: Emerg Infect Dis. 2019 Jul;25(7):e181421. doi: 10.3201/eid2507.181421 (PMC6590736; doi:10.3201/eid2507.181421)
Supplement: Appendix 1 — Overview of approaches to collecting mobility data for mapping infectious disease outbreaks, including information on smartphones, global positioning system trackers, and call detail records. [file 18-1421-Techapp-s1.pdf]

Article DOI: https://doi.org/10.3201/eid2507.181421

Ethical Considerations for Movement Mapping to Identify Disease Transmission Hotspots

Appendix 1

Overview of mapping approaches, including information on smartphones, global positioning system (GPS) trackers, and call detail records

Smartphones

Smartphones can either be equipped with an application that tracks global positioning system (GPS) signals or requests permission to retrospective mobility data already gathered by other currently installed apps. An alternative is Google Location History, which is passively gathered on Android phones over long periods of time and has an accuracy similar to GPS in the UK (*1*,*2*). The advantage is that detailed movement signals will be available. The downside of this approach is that smartphone use, especially in low-income countries, is strongly associated with socio-economic status, limiting participation of the poorer patients mostly affected by infectious diseases (*3*–*5*). Also, the common practice of sharing a phone can limit the accuracy of data collected in such contexts. In low-endemic, high-income countries, such as those in the EU, due to greater smartphone penetration, this approach is likely more feasible and informative than the use of CDRs. However, poverty might again limit smartphone use in those at highest risk for certain infectious diseases.

Global Positioning System (GPS) Trackers

Alternatively, a participant can be asked to carry a GPS tracker during a certain period. Different designs are available that can be easily carried and will record the study participant’s position every few minutes. Moreover, unlike in the case of smart phones, GPS trackers give strong control over access to the data, which minimizes the confidentiality and privacy risks to the participant, simply by leaving the device at home when they do not wish to be tracked. Disadvantages of handing GPS trackers to patients with a diagnosed infection include the

assumption that the prospectively collected movements are not modified according to the illness, diagnosis, or treatment. Especially in the presence of an ongoing outbreak the prospectively collected movements might vastly differ from the patient's movements during the height of infectiousness. Moreover, the GPS tracker can be unintentionally forgotten at home, and from a disease surveillance perspective, the increased level of privacy for the participants can also be a disadvantage as these missed locations can be of high epidemiologic interest.

Call Detail Records

Mapping individual movements in a population has become technically feasible by using call detail records (CDRs) systematically collected by telecom operators (*6*). CDRs consist of digitalized and organized information generated each time a mobile phone is used, e.g., calling, texting, connecting to mobile internet, and charging prepaid credit. CDRs include attributes such as a timestamp, source number, destination number, and most importantly for mobility mapping, the telephone mast (cell site) position showing the approximate geographic location. The spatial signal is less precise than what can be obtained by GPS tracking, as it results from assignment of the user to a telephone mast that is routing the call or text. The resolution is higher in urban areas (up to 50–100m in resolution) than in rural areas where fewer masts are placed.

A population-level analysis would benefit from the inclusion of all phone companies. While limited to active data points, no spatial information is available in the CDRs when the phone is not in use, the signal could be enhanced by sending short message service (SMS) messages to these targeted participants, generating an active data point for recording. An advantage is that mobile network operators typically maintain records for at least 3 months, allowing retrospective analysis of the period before transmission was interrupted by treatment, or the period before an outbreak is declared, and that this method is scalable to larger populations (*7*). Disadvantages include that persons of lower socio-economic status, children, and elderly persons might be underrepresented in the analyses, the lower spatial resolution in remote rural areas, and that details on phone sharing and double subscriber identity module (SIM) card use would need to be captured.

The use of CDRs provided by mobile network operators opens the possibility to map movements of large numbers of people (*5*,*6*,*8*), although expectations do not necessarily translate to impact (*9*). For instance, in Senegal mobile phone data of ≈150,000 users were used to build

an epidemiologic model that highlighted the effect of mass gatherings on the spread of cholera in the country (*10*). A major hurdle associated with the potential utilization of CDRs for disease control purposes is that a third party, in this case for-profit mobile network operators, is involved in the research project. Conversely, from the mobile network operator perspective, this is data that they already collect, whether a study is happening or not. For them the ethical concern is that this data are shared with a third party, i.e. the researchers. This sharing of CDRs potentially increases the risks to the individual of confidentiality breaches. Mobile network operators in turn reflect on ethical aspects of public health or medical research use of CDRs (*11*).

References

1. Ruktanonchai NW, Ruktanonchai CW, Floyd JR, Tatem AJ. Using Google Location History data to quantify fine-scale human mobility. Int J Health Geogr. 2018;17:28. PubMed http://dx.doi.org/10.1186/s12942-018-0150-z

2. Wesolowski A, Eagle N, Noor AM, Snow RW, Buckee CO. The impact of biases in mobile phone ownership on estimates of human mobility. J R Soc Interface. 2013;10:20120986. PubMed http://dx.doi.org/10.1098/rsif.2012.0986

3. Wesolowski A, Stresman G, Eagle N, Stevenson J, Owaga C, Marube E, et al. Quantifying travel behavior for infectious disease research: a comparison of data from surveys and mobile phones. Sci Rep. 2014;4:5678. PubMed http://dx.doi.org/10.1038/srep05678

4. Jones KH, Daniels H, Heys S, Ford DV. Challenges and potential opportunities of mobile phone call detail records in health research: review. JMIR Mhealth Uhealth. 2018;6:e161. PubMed http://dx.doi.org/10.2196/mhealth.9974

5. Wesolowski A, Buckee CO, Engø-Monsen K, Metcalf CJE. Connecting mobility to infectious diseases: The promise and limits of mobile phone data. J Infect Dis. 2016;214(suppl_4):S414–20. PubMed https://doi.org/10.1093/infdis/jiw273

6. Erikson SL. Cell phones—self and other problems with big data detection and containment during epidemics. Med Anthropol Q. 2018;32:315–39. PubMed http://dx.doi.org/10.1111/maq.12440

7. Finger F, Genolet T, Mari L, de Magny GC, Manga NM, Rinaldo A, et al. Mobile phone data highlights the role of mass gatherings in the spreading of cholera outbreaks. Proc Natl Acad Sci U S A. 2016;113:6421–6. PubMed http://dx.doi.org/10.1073/pnas.1522305113

8. Sangokoya D, Letouzé E, Data-Pop Alliance. How to use big data? Leading experts' roadmap to data-driven innovation projects. Vodafone Institute for Society and Communications, editors. Berlin: Vodafone Institute; 2017 [cited 2019 Feb 7]. https://www.vodafone-institut.de/wp-content/uploads/2017/11/How-to-use-Big-Data_VFI_Data-Pop-Alliance-Paper.pdf

9. The National Commission for the Protection of Human Subjects of Biomedical and Behavioral Research. The Belmont Report: ethical principles and guidelines for the protection of human subjects of research. Pub. no. 78-0012. Bethesda (MD): The Commission; 1979.

10. European Union General Data Protection Regulation (GDPR). Frequently asked questions about GDPR [cited 2019 Feb 7]. https://www.eugdpr.org/gdpr-faqs.html

11. Council for International Organizations of Medical Sciences (CIOMS). International ethical guidelines for health-related research involving humans. 4th edition. Geneva: The Council; 2016.
