# Supplementary material for: Ethical Considerations for Movement Mapping to Identify Disease Transmission Hotspots
Source: Emerg Infect Dis. 2019 Jul;25(7):e181421. doi: 10.3201/eid2507.181421 (PMC6590736; doi:10.3201/eid2507.181421)
Supplement: Appendix 2 — Background, objective, participants, and agenda for the 1-day workshop on the ethical aspects on the use of individuals’ mobility data for mapping infectious diseases held October 24, 2017, at the Institute of Tropical Medicine, Antwerp, Belgium. [file 18-1421-Techapp-s2.pdf]

Article DOI: https://doi.org/10.3201/eid2507.181421

Ethical Considerations for Movement Mapping to Identify Disease Transmission Hotspots

Appendix 2

Background, objective, participant's backgrounds, and agenda for the 1-day workshop on the ethical aspects on the use of individuals' mobility data for mapping infectious diseases held October 24, 2017, at the Institute of Tropical Medicine, Antwerp, Belgium

Workshop on the Ethics Surrounding Phone Tracking of Infectious Diseases

Background:

In the context of the development and review of the TB Enhanced Place Finding project, multiple ethical challenges were identified around the use of phone signals to see where tuberculosis (TB) patients crossed in time and space as a novel approach to identifying transmission hotspots.

Objective:

By inviting key experts and stakeholders, we aim to reflect on the risks and benefits of using tracking approaches by phone, global positioning system (GPS) signal, or otherwise for identification of transmission hotspots of TB and other infectious diseases, and on potential ways to reduce the risk. We plan to distil from this workshop an outline of a position paper that addresses challenges and solutions and ethical standards to consider when undertaking such efforts.

Participants:

- Ethicists, 2
- Scientists, 8, fields include epidemiology, bioinformatics, molecular biology, anthropology
- Information and communication technology experts, 2

- Data security specialists, 2
- Big data expert, 1
- Representatives of the Gambia Government and Medical Research Council joint ethics committee, 2
- Gambian National Leprosy and TB Programme representative, 1
- Expert on health, law, information technology, mobility data sharing, 1
- e-Health software provider, 1

Agenda

Part 1: Technicalities of phone tracking

9:10–9:30 The possibilities of phone tracking: TB phone tracking project in the Gambia, Enhanced Place Finding proof-of-concept project as introduction, to outline stakeholders and ethical challenges

9:30–9:50 Alternative approaches, such as global positioning system trackers: potential benefits and downsides

10:00–10:20 Phone tracking technology briefly described

10:30–10:50 The importance of informed consent and confidentiality agreements between all parties

11:10–11:30 Comprehension of the informed consent

11:40–12:00 The use of phone data from the perspective of an African regulator: How to protect consumer rights and government interests

12:10–12:30 The use of phone data from the legal and ethical perspective in Africa

12:30–13:00 Encryption, hashing and protection of privacy, Data Protection Impact Assessments (DPIAs) and General Data Protection Regulation (GDPR, EU Regulation 2016/679), a European perspective

Part 2: The future

14:00–14:30 Developing the app: technical and ethical challenges with implementation

14:30–14:45 Bigger picture, application for hotspot detection in other infectious diseases

14:45–15:30 Broader discussion

- Stigmatisation in infectious diseases

- Expand to other diseases: specific ethical challenges of phone tracking around
  - Sexually transmitted diseases/HIV
  - Malaria
  - Ebola....
- Using internet data rather than call detail records: technical and ethical challenges
- Could phone tracking be used in Europe, Asia, America, or other locations?

Part 3: Key aspects to be summarized in a position paper

15:50–16:50 Sum up and address the identified challenges, aiming for an outline of a position paper that addresses ethical challenges and solutions for phone tracking of infectious diseases.
