# Supplementary material for: Ethical Considerations for Movement Mapping to Identify Disease Transmission Hotspots
Source: Emerg Infect Dis. 2019 Jul;25(7):e181421. doi: 10.3201/eid2507.181421 (PMC6590736; doi:10.3201/eid2507.181421)
Supplement: Appendix 3 — Data protection impact assessment template developed by White Wire Data Protection, Kontich, Belgium. [file 18-1421-Techapp-s3.pdf]

Article DOI: https://doi.org/10.3201/eid2507.181421

Ethical Considerations for Movement Mapping to Identify Disease Transmission Hotspots

Appendix 3

Data Protection Impact Assessment template developed by White Wire Data Protection, Kontich, Belgium (following pages)

Data Protection Impact Assessment

Document Implementation of DPIA For XXX

Performed by:	
Last update:	
Date of advice Data Protection Officer:	
Date Approved Directorate/Ethics Committee:	

WHITE WIRE

1 Contents

1 Contents 2

2 Management Summary 4

3 Framework 5

3.1 Context Organization 5

3.2 Context processing /Project 5

3.3 Project Planning 5

4 DPIA Project Xxx 6

4.1 Scope 6

4.2 Relevant codes of conduct or certifications 6

4.3 (Joint) Controller 6

4.4 Concerned Actors 6

4.5 Categories of persons and personal data 6

4.5.1 Categories of data subjects 6

4.5.2 Categories of Personal data 6

4.6 Description of the processing in detail 6

4.6.1 Step 1: e.g. Provinding information and gathering consent 6

4.6.2 Step 2 7

4.7 Processors and processor agreements 7

4.8 Compliance with basic principles for processing of personal data 7

4.8.1 Lawfulness, Fairness, and Transparency (information) 7

4.8.2 Purpose 7

4.8.3 Minimal data processing 7

4.8.4 Accuracy 7

4.8.5 Storage limitation 7

4.8.6 Integrity & Confidentiality 7

4.9 Rights of the data subject 8

4.9.1 Possibilities for exercising rights of the data subject 8

4.9.2 Right to Information 8

4.9.3 Right of Access 8

4.9.4 Right to Rectification 8

4.9.5 Right to Data Erasure 8

4.9.6 Right to limitation of processing 8

4.9.7 Right to portability 8

4.9.8 Right of objection 8

5 Risks 9

5.1 Risk methodology 9

5.2 Identified risks 9

6 Measures taken 10

6.1 RISK-001: Legal Position of Controller 10

6.2 RISK-004: Use National Identification number without permission 10

6.3 RISK-005: U.S. hosting provider 10

7 Residual risks 10
7.1 Overview residual risks 10
7.2 Advice from the DPO 11
7.3 Decision on prior consultation with DPA 11

2 Management Summary

This document documents a Data Protection Impact Assessment (DPIA) as described in the General Data Protection Regulation. This DPIA is an analysis of the intended processing of personal data for Project XX and contains the general context, information on the processing, assessment of the inherent and residual risks and concrete measures to be taken to mitigate these risks, and includes a formal advise on the need for prior consultation with a Data Protection Authority (DPA).

Further summary of the DPIA.

3 Framework

3.1 Context Organization

Describe the organization, its tasks and other information relevant in the context of the DPIA. This information serves to clarify why this organization intends to carry out the proposed processing.

3.2 Context processing /Project

General description of the project, for full details see 4.5.

Continuing on 3.1, how does this process connect to the context of the Organization.

3.3 Project Planning

If the DPIA is part of an ongoing project, describe the general planning and deadlines, including the time table of execution of the DPIA. This is especially useful in projects with a tight schedule where the performance of a DPIA should be carefully orchestrated.

4 DPIA Project Xxx

4.1 Scope

What is included and what is excluded in this DPIA. This can relate to specific aspects of research or to clarify which tools or processing have or have not been taken into account.

4.2 Relevant codes of conduct or certifications

Are there any codes of conduct or certifications relating to data protection applicable to the intended processing, or the sector of the controller?

Does the controller meet these codes of conduct/certifications?

4.3 (Joint) Controller

Who is the controller? Sometimes this is simply the organization itself, in other cases there may be partnerships where the determination of the respective responsibilities is an essential part of the DPIA to correctly document joint controllerships and the responsibilities it entails.

4.4 Concerned Actors

List of relevant organisations and persons incl. their function. Think of stakeholders such as the relevant data protection authorities, individuals (clients, patients, residents, etc), interest groups, the Data Protection Officer (DPO), processors, etc.

4.5 Categories of persons and personal data

4.5.1 Categories of data subjects

Whose personal data are processed within the scope of this DPIA? (Employees, patients, clients, residents, research participants, users, etc.)

4.5.2 Categories of Personal data

Which categories of personal data are processed? Include non-sensitive personal data (identification data, financial data, etc) as well as sensitive personal data (articles 9 and 10 GDPR)

4.6 Description of the processing in detail

By means of diagrams or simply text, present the data flow, from reception or creation of the data to eventual destruction, archiving or forwarding, also called the data life cycle.

This section should also establish whether there will be any forwarding of data to third countries and whether these third countries offer adequate protection (possibly by reference to other relevant parts in the DPIA).

4.6.1 Step 1: e.g. Provinding information and gathering consent

4.6.2 Step 2

4.7 Processors and processor agreements

Which processors are involved in the processing, and are appropriate agreements in place (processor agreements or DPA's (data processing agreements)) as described in article 28 of the GDPR?

4.8 Compliance with basic principles for processing of personal data

Given the information in 4.1 and 4.3, please specify compliance with the basic principles of processing.

4.8.1 Lawfulness, Fairness, and Transparency (information)

Describe the manner in which information is provided to the data subject and the legal basis for the processing of personal data with special attention to "legitimate interest".

If relevant, also describe in which way there has been communication with stakeholders or representatives, interest groups, and the request for their opinion.

4.8.2 Purpose

What are the clear, specifically defined purposes of data processing?

4.8.3 Minimal data processing

Given the Purpose mentioned in 4.8.2, which personal data is required? (Categories of data or effective listing of all data) Are only those data processed?

4.8.4 Accuracy

Is the data that is processed accurate and correct? (links to other data sources to keep data up to date, periodic pop ups for the user to review data, online portal for stakeholders, etc.)

4.8.5 Storage limitation

What is the retention period of the data, and why exactly that retention period? Sometimes this is laid down by law, sometimes it has to be substantiated by a specific justification.

4.8.6 Integrity & Confidentiality

How are confidentiality, integrity (and availability) of the data secured?

To test these criteria, the domains from the ISO27002 are used as the basis. Other standards or certificates obtained may also suffice as a justification for providing the appropriate measures to ensure the integrity, confidentiality and availability of data.

4.8.6.1 Security policies

4.8.6.2 Risk Analysis and remediation plans

4.8.6.3 Appointment of a DPO

4.8.6.4 Organization of Information security

4.8.6.5 Human Resource Security

4.8.6.6 Asset Management

4.8.6.7 Access Control (logical)

4.8.6.8 Cryptography

4.8.6.9 Physical security

4.8.6.10 Operational security

4.8.6.11 Communication security

4.8.6.12 System acquisition, development and maintenance

4.8.6.13 Supplier and processing relations

4.8.6.14 Security incident management

4.8.6.15 Business Continuity Management

4.8.6.16 Compliance & Accountability

4.9 Rights of the data subject

In the first instance, it is necessary to describe how a data subject can exercise his/her rights, i.e. via a telephone number that can be called, physical location to visit, email address to write to, etc., or any other way a data subject can exercise his or her rights (section 4.9.1).

The following sections address the different rights: are they applicable, when, and how is compliance assured (e.g. online portal, request by email according to process described in 4.9.1).

4.9.1 Possibilities for exercising rights of the data subject

4.9.2 Right to Information

4.9.3 Right of Access

4.9.4 Right to Rectification

4.9.5 Right to Data Erasure

4.9.6 Right to limitation of processing

4.9.7 Right to portability

4.9.8 Right of objection

5 Risks

Listing of detected risks without additional measures taken (inherent risk). In other words, we are now reviewing the situation based on the description in the previous chapters. What are the risks or problems we identify in relation to the data we process (e.g. the basic principles of personal data processing, security of the information), and in a broader context, the possible impact on persons whose data are processed (e.g. Rights of the individual, reasonable expectations, sensitivity of the data, possible consequences of a data leak).

In Short: once all previous chapters have been completed, there are most likely deviations or aspects that require further scrutiny. Examples are included below including an estimation of severity.

5.1 Risk methodology

How are the risks estimated or identified (through interviews, analysis of documentation, risk assessment criteria etc) and based on what criteria is a severity assigned to the risks? This requires a description of how risk is measured. Ideally these scores or measurements are objective: if person X performs a risk analysis and person Y performs the same analysis later, the same scores will be applied due to the objective nature of the scoring criteria.

5.2 Identified risks

Nr	Description	Severity	Chapter
RISK-001	Ambiguity: Joint processing officers or one processing officer, namely the Xxxx?	Low	4.2
RISK-002	Enter Xxxxx File number to copy data: How unique are these file numbers? Other ' acquisition of other file functionalities '?	Low	4.2
RISK-003	Data subjects are not adequately informed due to language barriers	Middle	4.3
RISK-004	Intended use of the National Identification number without the authorisation of the SC	Critical	4.6
RISK-005	Xxxxx uses U.S. hosting, and may also process the National Identification number	High	4.7
RISK-006	Retention periods have not yet been validated	Middle	4.8.5
RISK-007	Logging currently provided is not sufficient to track all create, read, update and delete (CRUD) actions on personal data (test to be validated)	Middle	4.8.6
RISK-008	Xxxx Does not possess a compliant Processing Agreement	High	4.8.6
RISK-009	Web application that is accessible to the public and will possibly process National Identification number : no penetration testing provided.	High	4.8.6
RISK-010	Default setting includes Transfer to XXX, does not adhere to data protection by default principles	High	4.8.1

6 Measures taken

Describe what measures have been taken to mitigate the risks as identified in 5.2. This usually involves reference to a risk number and a description of risk treatment (how has a risk been addressed). Risk treatment can involve for possible treatments (in order or preferred approach):

- Avoidance: ensure the risk cannot take place by completely eliminating it
- Transfer: the risk still exists, but is now the responsibility of someone else
- Mitigation: some residual risk may exist, but measures have been taken to mitigate the risk as much as possible
- Acceptance: no treatment except the realization that the risk can occur and no specific measures will be taken to reduce it (further).

6.1 RISK-001: Legal Position of Controller

6.2 RISK-004: Use National Identification number without permission

6.3 RISK-005: U.S. hosting provider

7 Residual risks

Analyse here which of the risks identified in Chapter 5 are not adequately covered by measures in Chapter 6. These are the residual risks. Below you will find an overview of various potential risks that cannot be covered by the various measures (i.e. residual risk).

7.1 Overview residual risks

No	Description	Ernst	Chapter
RISK-001	Lack of a privacy policy	Critical	4.1
RISK-002	Absence of an information security plan	Middle	4.2
RISK-003	Access control (logical) of application X is insufficient	High	4.3
RISK-004	Access control (logical) to file server has no assigned roles	Middle	4.6
RISK-005	No clearly defined or determined purpose	Critical	4.5
RISK-006	Processing agreements are not in place	Critical	4.7

7.2 Advice from the DPO

If appointed, it is mandatory to involve the DPO in the execution of the DPIA[1]. Concerning the analysis in this document, what advice does the DPO have regarding the processing (s) that forms part of the DPIA? Proceed with the processing? Specific residual risks that still need to be addressed? Is prior consultation with the Data Protection Authority warranted?

7.3 Decision on prior consultation with DPA

Depending on the residual risks from 7.1, it is stated here whether a prior consultation with the Data Protection Authority is required. In other words, whether the local data protection authority should be contacted to ask for advice on processing because the residual risks are (possibly) too high.

If relevant, include the approval or reference to a report from a project team, management consultation, etc. where the decision on the prior consultation has been discussed.

----------End of Document---------

1 Art 35.2 GDPR
